# Assessment of Volume Status During Prone Spine Surgery via a Novel Point-of-care Ultrasound Technique

**DOI:** 10.7759/cureus.4601

**Published:** 2019-05-05

**Authors:** Jeremy Hensley, Hong Wang

**Affiliations:** 1 Anesthesiology, Ruby Memorial Hospital, Morgantown, USA; 2 Anesthesiology, West Virginia University, Morgantown, USA

**Keywords:** point-of-care ultrasound, spine surgery, prone position, inferior vena cava, volume assement

## Abstract

Background

Operations performed with the patient in the prone position can pose a significant challenge to the anesthesiologist. Hypotension is a commonly encountered complication. Intravascular volume depletion and decreased cardiac output secondary to decreased preload are thought to be the most likely cause of hypotension in the prone position. Measurement of inferior vena cava (IVC) diameter via point-of-care ultrasound examination (POC_US) has been used to provide an estimate of intravascular volume status. However, this measurement is most often obtained with the patient in the supine position.

Materials and methods

In this study, we describe a technique for evaluating IVC diameter via POC_US in the prone position. Right lateral long axis imaging of the IVC was used to assess the intravascular volume status of 10 patients undergoing lumbar spine surgery in the prone position. In addition, we used a non-invasive measure of cardiac output to correlate changes in IVC width with changes in cardiac output.

Results

Images of the IVC in the prone position were obtainable in all 10 patients. IVC diameter increased in six out of 10 patients on going from supine to prone position. The increase in IVC diameter corresponded to an increase in cardiac output, measured noninvasively in five out of the six patients.

Conclusions

Our findings indicate that POC_US examination of the IVC is possible in the prone position. Further study of a larger patient population could demonstrate the utility of this technique in assessing intravascular volume status in patients undergoing surgery in the prone position.

## Introduction

Hypotension in prone patients is a common problem encountered by anesthesiologists in the operating room. The reasons for this are likely multifactorial, but decreased stroke volume and cardiac output are the most commonly cited causes. Hypovolemia can exacerbate the hypotension phenomenon. One study has demonstrated a 24% decrease in the cardiac index when going from the supine to the prone position [1]. These hemodynamic derangements are likely due to decreased venous return and ventricular compliance. A study using transesophageal echocardiography suggests that the optimization of intravascular and the use of the Jackson frame may be useful in preventing and treating this condition [2]. Prevention of hypotension in the prone position is of great importance, given the association with potentially devastating complications such as perioperative visual loss.

Point-of-care ultrasound (POC_US) has been applied in perioperative medicine [3-4] Its examination of the inferior vena cava (IVC) can assist in predicting the volume status. Hypovolemic patients presenting to the emergency department have consistently smaller IVC diameters as compared with ones in euvolemic patients [5]. In addition, hypovolemic patients undergoing hemodialysis show an exaggerated collapse response during respiratory cycles [6]. It has also been reported that the IVC diameter in combination with low mean arterial pressure on admission can predict long-term mortality in patients admitted to the hospital for acute heart failure [7].

However, the IVC diameter is affected by several other factors such as intra-abdominal pressure and right atrial pressure [8-9]. A patient’s position may also affect the diameter of the IVC. For example, authors have demonstrated that IVC dimensions are larger in the supine position as compared with ones in the left lateral position [10].

Several techniques for acquiring views of the IVC have previously been described, with varying rates of inter-rater reliability. One study showed that inter-rater reliability was highest for B-mode measurement of IVC diameter using the sub-xiphoid transabdominal long-axis view and lowest when using M-mode to calculate collapsibility indices [11].

The right lateral ultrasound view has been successfully used to estimate the diameter of IVC in pregnant women [12]. American Society of Echocardiography guidelines suggest that IVC is best assessed via the subcostal window in supine and spontaneously breathing patients [13]. However, previous studies have shown that changes in IVC diameter may be useful in predicting fluid responsiveness in mechanically ventilated patients as well [14].

In this brief report, we describe a novel technique for measuring IVC diameter in the prone position and attempt to correlate changes in the IVC diameter with the noninvasive measurement of cardiac index in patients undergoing lumbar spine surgery.

## Materials and methods

In all patients, standard induction was performed with fentanyl, propofol, and rocuronium, with dosing left to the discretion of the attending anesthesiologist. All patients were then intubated via direct laryngoscopy. Anesthesia was maintained with sevoflurane and analgesic medications chosen by the attending anesthesiologist. The patients were carefully placed in a prone position by operating room staff. All patients were extubated at the completion of surgery.

**Figure 1:**
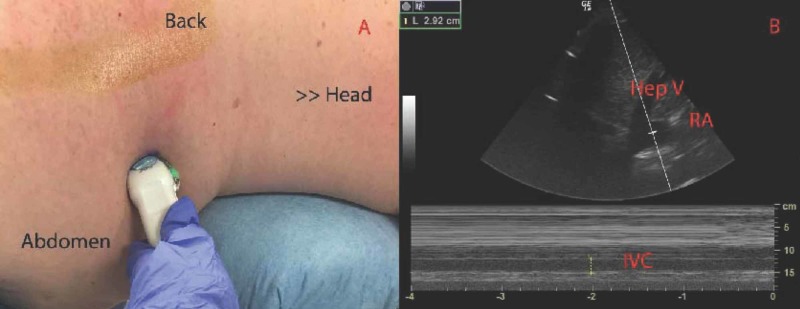
Right lateral long axis IVC probe position (A) and VC imaging (B) Hep V: Hepatic Vein. RA: Right Atrial. IVC: Inferior Vena Cava

The ultrasound images were obtained with LOGIQe echography (GE Healthcare, Illinois, United States). The 3S-RS probe with abdomen setting was placed at the patient’s right upper abdomen mid axillary line. Right lateral long axis IVC images were obtained from 10 patients (Figures 1A-1B). To avoid the discrepancy in IVC changes between spontaneous breathing and mechanical ventilation, all images were obtained during mechanical ventilation. The specific time points included: after induction/intubation, supine position (Supine); before incision, prone position (Prone 1); and after closing, prone position (Prone 2). The diameter of the IVC was measured at the junction of the IVC and hepatic vein with M-mode (Figure 1B). All ultrasound examinations were completed by an expert in IVC ultrasounds. All patients were hemodynamically stable and had no support with any vasoactive agents at the time point of ultrasonography. The compressibility index (CI) was not measured. This is due to the fact that CI is not reliable and applicable during mechanical ventilation.

ClearSight (Edwards Lifesciences Corp, Irvine, CA, US) is a continuous noninvasive hemodynamic monitor that uses a volume clamp and physical methods. We further used ClearSight Volume View to validate the changes in volume status obtained by the ultrasonographic measurement of the IVC. The ClearSight data were obtained at the time of the ultrasonography. The ClearSight data were blind to the person who was performing the ultrasound measurement.

## Results

We successfully obtained ultrasound images of all 10 patients in the prone position. Figures 1A-1B show the probe position and image acquisition.

To evaluate the diameter of the IVC in different positions, we compared the diameters of the IVC in the supine and prone positions. We found that the diameters increased with time in six of the 10 patients. This increase was not associated with the patient’s body mass index (BMI). We further evaluated if the ultrasound exam of the IVC in the prone position can be validated by other non-invasive monitors. We used ClearSight Volume View in patients #4 to #10. We found that the diameter of IVC increased in five of the six patients over the course of surgery, which corresponded to increases in cardiac index measured by using ClearSight Volume View (Table 1).

**Table 1:** Comparison of diameters Supine: After induction and intubation Prone 1: Prone position before the incision Prone 2: Prone position after the closing CI1: Cardiac index of Prone 1 CI2: Cardiac index of Prone 2

	Supine	Prone 1	Prone 2	CI1	CI2
#1	2.1	2.5			
#2	1.7	2.1			
#3	1.9	2.1			
#4	1.8	2.2			
#4	2.0	2.0	2.3	2.1	3.4
#5	1.8	2.0	2.0	2.3	2.0
#7	1.8	2.0	2.1	2.8	3.4
#8	2.2	2.2	2.7	4.0	5.8
#9	2.0	2.0	2.1	2.8	4.1
#10	2.35	2.35	2.92	2.7	4.3

## Discussion

Measurement of the IVC via POC_US for guiding intravascular volume resuscitation is traditionally performed in the supine position. The most commonly used view is the subcostal view in the sagittal plane. Although the subcostal view is likely superior, a prior study has shown that the right lateral long axis view may be used [15]. Because the subcostal view is impossible to obtain in the prone position, we have attempted to demonstrate the utility of the right lateral long axis view in assessing intravascular volume status in prone patients. To our knowledge, there are no previous studies documenting the use of IVC POC_US in assessing intravascular volume status in the prone position.

The right lateral long axis IVC ultrasound view was compared with the subcostal view in a previous study [16]. The right lateral long axis view may underestimate the changes in volume status as compared to the subcostal view. This is due to the fact that the IVC may collapse and expand to a different degree along its anterior-posterior and medial-lateral axes. The right lateral view measures the medial-lateral diameter while the subcostal view measures the anterior-posterior diameter. In our study, we used the right lateral view to measure the IVC diameter in both supine and prone positions. We further used ClearSight to validate that the medial-lateral diameter changes are consistent with the patient’s volume status.

A previous study attempted to evaluate the IVC diameter in the prone position using the subcostal view in healthy volunteers [17]. The diameter of the IVC was correlated to the abdominal pressure and weight force of the upper thorax. The diameter increased when the position was changed from the standing to the prone position. No comparison was done on diameter changes from the supine to the prone position. In addition, the study was done with volunteers on a flat, hard table that had a 10 cm opening at the upper abdomen/lower chest to facilitate ultrasound examination. In our study, the patients were on the Jackson table. The Jackson table allows the abdomen to hang freely, resulting in decreased compressive forces and effects on hemodynamic parameters when compared to other prone operating tables. Our study indicated that the IVC diameter increased with the position changes from the supine to the prone position. This may be due to less compression of IVC during the prone position with a free-hanging abdomen. Several studies had shown that the collapsibility index (CI, IVC max - IVC min/IVC max) during spontaneous respiration could predict the volume status and correlate with central venous pressure (CVP). CI measurement was not reliable and applicable during mechanical ventilation15. The study presented here is conducted entirely during mechanical ventilation. Therefore, CI was not measured.

We have demonstrated that the right lateral view of the IVC is obtainable in the prone position. However, it is difficult to interpret the utility given the small number of patients in the study. In several of our patients, an increase in the diameter of IVC corresponded to an increase in cardiac index measured non-invasively, which potentially signifies ongoing fluid resuscitation. Thus, one might speculate that trending changes in IVC width might be an effective means of guiding resuscitation in prone surgery. In conclusion, the measurement of IVC width with point-of-care ultrasound using the right lateral long axis view is possible in the prone position. Further study of a larger patient population can potentially demonstrate the utility of this technique in assessing intravascular volume status in patients undergoing surgery in the prone position.

## Conclusions

Our findings indicate that POC_US examination of the IVC is possible in the prone position. Further study of a larger patient population could demonstrate the utility of this technique in assessing intravascular volume status in patients undergoing surgery in the prone position.
